# Association between cognitive health and masticatory conditions: a descriptive study of the national database of the universal healthcare system in Japan

**DOI:** 10.18632/aging.202843

**Published:** 2021-03-19

**Authors:** John D. Da Silva, Shy Chwen Ni, Cliff Lee, Hawazin Elani, Kailing Ho, Carlos Thomas, Yukinori Kuwajima, Yoshiki Ishida, Takuya Kobayashi, Shigemi Ishikawa-Nagai

**Affiliations:** 1 Restorative Dentistry and Biomaterial Sciences, Harvard School of Dental Medicine, Boston, MA 02115, USA; 2 Oral Medicine, Infection and Immunity, Harvard School of Dental Medicine, Boston, MA 02115, USA; 3 Oral Health Policy and Epidemiology, Harvard School of Dental Medicine, Boston, MA 02115, USA; 4 DMD Candidate, Harvard School of Dental Medicine, Boston, MA 02115, USA; 5 Iwate Medical University, School of Dental Medicine, Japan

**Keywords:** mastication, cognitive decline, dementia, Alzheimer’s disease, descriptive study

## Abstract

Cognitive health is subject to decline with increasing numbers of lost teeth which impacts mastication. This study is a descriptive data analysis of the association between masticatory and cognitive conditions using a large database. We obtained the dental and medical records from Japan's universal healthcare system (UHCS) from the national database in 2017. The data from 94% of the Japanese population aged 65 and over is included. It is inclusive of diagnostic codes for various types of cognitive impairment, as well as dental treatment records from 2012 to 2017. The cognitive impairment group was compared to those without a diagnosis of cognitive impairment. Crude odds ratio between loss of mastication with natural teeth (exposure) and cognitive impairments (outcome) were compared. Patients who have lost masticatory function are likely to have cognitive impairment with an odds ratio of 1.89 (p<0.0001) for early elderly (aged 65-75) and 1.33 (p<0.0001) for advanced elderly (over 75). Patients who are edentulous and function with complete dentures are likely to have cognitive impairment with an odds ratio of 2.38 (p<0.0001) and 1.38 (p<0.0001), respectively. The data shows a convincing and significant result of an association between cognitive health and oral health, related to masticatory conditions.

## INTRODUCTION

Dementia is a degenerative disease characterized by progressive deterioration in mental abilities affecting behavior, cognition, and function. Alzheimer’s disease (AD) is the leading cause of dementia in the geriatric population and the fifth leading cause of death in people over the age of 65 in the US [1]. With the aging population growing as people live longer, globally up to 131.5 million people are projected to be diagnosed with dementia by 2050 [1, 2]. Age related chronic disease has become a global health concern and financial burden that impact individuals with dementia, their family and friends, as well as society as a whole.

The pathologic hallmarks of AD, based on autopsy, are characterized by aggregates of extracellular β-amyloid peptide (Aβ), and intracellular neurofibrillary tangles of tau protein. In addition to pathophysiologic mechanisms, recent studies have focused on factors that lead to neuronal damage prior to cognitive decline and prevention strategies using physical and/or cognitive training. For example, participation in leisure activities, playing board games, musical instruments, or social network activity are essential factors for the maintenance of cognitive health [3–5]. Regular exercise and dietary intake of antioxidants or foods rich in anti-inflammatory components are also believed to help maintain cognitive function [6–9]. These preventative measures are additionally beneficial to improve comorbid conditions that accelerate brain aging in AD patients such as cardiovascular disease, and diabetes [10, 11].

In the field of dentistry, several studies have investigated the relationship between oral function and cognitive function. Tooth loss effects spatial memory and increases the risk of diminished cognitive function suggesting that reduced masticatory function may be a risk factor for dementia [12–14]. Animal studies indicate that reduced masticatory ability due to missing molars leads to impaired spatial memory and degeneration of hippocampal neurons [15–17]. A study of 16 patients with mild Alzheimer’s disease (AD) had reduced masticatory and cognitive function when compared to healthy non-AD patients [13]. Another study indicated that the risk of developing AD and dementia was 1.63 times greater in the elderly with no teeth when compared to those with 20 or more teeth [14]. A longitudinal study found that the higher the number of teeth lost, the faster cognitive function declined [18]. Though an inverse association between the number of remaining teeth and cognitive function was suggested, the subjects of these studies were either of a small sample or from a limited geographic area.

Rising evidence on the effects of chewing in increased attention, memory, and cognitive processing demonstrates that masticatory condition has a link to cognitive health [19]. Since exosomes can translocate from muscle cells to nerve cells as cargo vehicles, our hypothetic mechanism for connecting mastication and cognitive health is that muscle activity can directly affect amyloid β burden. In our recently published study [20, 21], we tested our hypothesis that muscle cells can package neprilysin in exosomes in response to a neuromuscular signal and retrograde axonal transport to the brain. Neprilysin (NEP), a member of metalloproteases, aids with clearance of Aβ, plays another role in the pathogenesis of AD and serves as molecular marker of disease [22]. *In vitro* experiments, cholinergic stimulation of myotube cells increases quantities of both the exosome secreted into the culture medium and the amount of exosomal NEP. In the presence of carbachol, a cholinergic stimulant, muscle cytosolic NEP decreased while exosomal-NEP secretion increased, which proved that exosomes packaged and secreted NEP protein in muscle cells. *In vivo* experiments, fluorescent labeled myotube cells derived exosomes were injected into masseter muscle of euthanized mice and were found in both trigeminal and hippocampal tissue lysate. Results showed that the hippocampus and cerebrum contained NEP protein in the absence of mRNA, which suggested that NEP is not native to the brain, and NEP is transported into the brain via retrograde axonal transport. It was also confirmed that transgenically expressed NEP was transferred to the hippocampus via the trigeminal nerve. If these NEP rich exosomes were able to gain access to the brain via retrograde transport, then there is the possibility that masticatory activity could contribute to reducing the Aβ plaque burden in the brain. The trigeminal nerve is the largest cranial nerve, and it innervates the masseter muscles, which are the strongest among the muscles of mastication. This anatomical fact led to our tested hypothesis that masseter muscles send exosomal NEP to the brain in response to cholinergic stimulation during chewing, thus bypassing the blood-brain barrier (BBB).

These findings demonstrate that a novel mechanism for the connection of the oral cavity to the brain exists. Neuroprotective agents synthesized by the muscles of mastication on stimulation can travel to the brain [20, 21]. These findings then led us to generate the current hypothesis that a lack of mastication with natural teeth, due to tooth loss, may negatively affect cognitive health. The field of Prosthodontics supports this hypothesis based on the determinants of maximally attainable bite force, which is an increased bite force with an increased number of teeth and occlusal tooth contacts [23]. The masticatory/chewing forces generated by a removable prosthesis is 30~60% of what the natural dentition is and 10~40% of it for compete dentures. Other studies also indicate that when the function of the masticatory system is reduced due to loss of occlusal support, removable prostheses do not compensate sufficiently to attain the previous maximum bite force [24–26].

Here, we report oral function, especially masticatory function, is associated with cognitive function in patients with Alzheimer’s’ disease and other dementias in a Japanese population. Japan has a Universal Healthcare System (UHCS) where medical and dental care are recorded together. In 2011, the Japanese government, Ministry of Health, Labor and Welfare established that third parties can access the UHCS database under government guidelines. Our global team was the first dental research group whose research proposal was approved to assess the association between mastication and cognitive impairment. This study is a descriptive data analysis to examine if there is an association between cognitive function and masticatory conditions, which we hope will lead to future case-controlled or cohort studies.

## RESULTS

### Data categorized based on age and cognition

The number of the patients over 65 who were recorded in the UHCS in 2017 was 33,191,456, which is approximately 94% of the entire population of those over 65 in Japan (Table 1). The 65-74 group included 15,661,136 patients and the 75 and older group included 17,530,320, which is about 88% and 99% of the population in those age categories, respectively. The data was then subcategorized into the cognitive impairment group (CI) and cognitive health control group (CH). 56,081 were diagnosed as CI in the 65-74 age group and 662,691 for the 75 and older group. The number of the patients who received dental care within six years from 2012~2017 was 38,333 (68.4%) in the CI group and 11,216,434 (71.9 %) in the CH group for the early elderly, and 432,754 (65.3%) and 11,521,764 (68.3%), respectively, for the advanced elderly (Table 1).

**Table 1:** Number of patient data extracted from Japanese universal healthcare system (UHCS) database.

**Number of patients recorded in medical-UHCS database in 2017**		**Age >65: 33,191,456 (about 94% of the population)**			
		**Age 65~74: 15,661,136 (88%)**		**Age >75: 17,530,320 (99%)**	
Cognitive diagnosis in 2017 • CI: Cognitive impairment • CH: Cognitive healthy (Control)		CI	CH	CI	CH
		56,081	15,605,055	662,691	16,867,629
Number of patients recorded in dental UHCS database within 2012~2017		38,333 (68.4%)	11,216,434 (71.9%)	432,754 (65.3%)	11,521,764 (68.3%)

### Distribution of masticatory classification in CI and CH group

**Figure 1:**
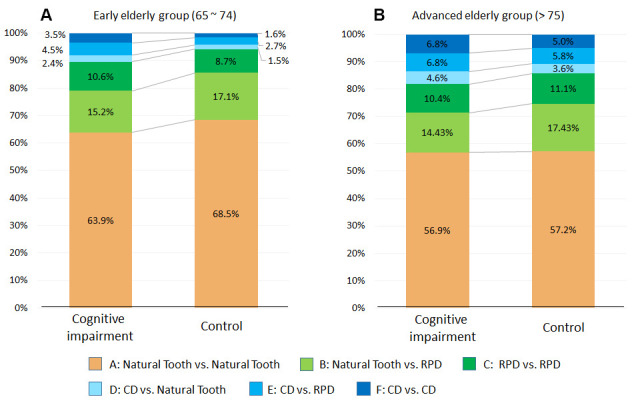
**Comparison of proportion of mastication patterns of cognitive impairment and control group.** Proportions of mastication patterns were shown between patients with CI and Control. Mastication pattern is further separated into early elderly group (ages 65 ~ 74) (**A**) and advanced elderly group (ages > 75) (**B**). 1/3 of all CI subjects had mastication patters with no teeth contact.

Table 2 represents the number of patients for each masticatory scheme for both CI and CH group. The proportions of masticatory patterns in CI group were compared with CH group as demonstrated in Figure 1. For the early elderly (ages 65~74), 94.2% of the control group exhibited all or some natural tooth-tooth contact (classification A, B or C), and only 5.8% exhibited no natural tooth-tooth contact (classification D, E or F). In contrast, in CI group, 89.7% exhibited all or some tooth-tooth contact and 10.4% presented with no natural tooth-tooth contact (Figure 1A). In the advanced elderly group (ages 75 and over), fewer subjects had all or some natural tooth-tooth contact: 81.8% for CI group and 85.7% for control group. Furthermore, 18.2% for CI group and 14.3% for control group presented with no natural tooth-tooth contact (Figure 1B).

**Table 2:** The number of patients in each mastication classification.

**Mastication Classification**	**Age 65~74**		**Age >75**	
	CI	CH	CI	CH
A	24,441	7,668,684	245,818	6,575,863
B	5,805	1,913,530	62,326	2,004,386
C	4,045	969,844	45,037	1,271,929
D	904	166,169	19,888	413,731
E	1,724	302,582	29,367	661,514
F	1,339	176,887	29,352	569,546

### Association between masticatory patterns and cognitive condition

Table 3 shows the crude odds ratio between loss of mastication in the absence of natural tooth contacts (exposure) and cognitive condition (outcome). Looking at mastication with natural tooth contact (exist: pattern A+B+C, absence: pattern D+B+F), patients who have lost natural tooth mastication are likely to have CI with an odds ratio of 1.89 (p<0.0001) for the early elderly group and 1.33 (p<0.0001) for the advanced elderly group. Looking at typical patterns A (all-natural tooth contacts) and F (all denture tooth contacts, CD in the Maxilla and Mandible), patients with only denture tooth contacts are likely to have CI with an odds ratio of 2.38 (p<0.0001) for the early elderly group and 1.38 (p<0.0001) for the advanced elderly group.

**Table 3:** Crude odds ratio between loss of mastication in the absence of natural tooth contacts (exposure) and cognitive condition (outcome).

	**All or some natural tooth contact (A,B,C)** **vs.** **No natural tooth contact (D,E,F)**		**All natural tooth contact (A)** **vs.** **All denture tooth contact (F)**	
**Age group**	**Early elderly**	**Advanced elderly**	**Early elderly**	**Advanced elderly**
Odds ratio	1.89	1.33	2.38	1.38
95% CI	1.8294 to 1.9541	1.3227 to 1.3437	2.2475 to 2.5099	1.3604 to 1.3946
Significance	*P* < 0.0001	*P* < 0.0001	*P* < 0.0001	*P* < 0.0001

## DISCUSSION

In the cognitive impairment group for both age groups, a higher percentage of patients exhibiting no natural tooth-tooth contacts (Classification D+E+F) was identified and compared to the control group (Figure 1). This reveals that individuals with signs of cognitive deterioration at the time of data collection had reduced masticatory function with the loss of some or all-natural tooth-tooth contacts. On the other hand, individuals with no signs of cognitive deterioration, at the time of data collection, had better masticatory function with more natural tooth-tooth contacts. This data is in consistent with the findings that a lack of posterior occlusion predicted the incidence of cognitive decline [27, 28]. Our result is additionally convincing and significant because the dataset consists of a large sample size, where almost all of the dental and medical treatments within one healthcare system were recorded and analyzed and not data from self-reports or surveys.

In mouse model studies, evidence suggests the negative effects of masticatory deficiency and tooth loss on neurogenesis, present as a risk factor for cognitive dysfunction [29, 30]. A systematic and meta-analytic review also concludes a 20% higher risk for developing cognitive decline in individuals with suboptimal dentitions (<20 teeth) and draws the links between oral and general health [12]. Though the mechanism of this relationship is not clearly understood, studies have found that during mastication, there is an increase of cortical blood flow in somatosensory cortical areas as well as increased oxygen levels in the prefrontal cortex and hippocampus [31]. The neurovascular aspects of AD pathophysiology, cerebral hypoperfusion and reduced glucose transport progresses to cognitive decline and hippocampal atrophy in the early stages of AD [31]. A most recent MRI study also showed significantly smaller total gray matter volume and regional reduction of gray matter in the left hippocampus and parahippocampus associated with an increased number of missing teeth in subjects with mild cognitive impairment of Alzheimer’s disease [32]. Moreover, a 9-year longitudinal study found that individuals with complete or partial tooth loss had significantly lower total brain volume and gray matter volume [33].

A possible explanation for the mechanism relating the brain to the oral cavity is that cranial nerves bypass the blood-brain barrier. This is possible via the trigeminal nerve which innervates the masseter muscles. The masseter muscles are responsible for chewing and forming food boluses for swallowing with masticatory movement generated by the neuronal network in the brainstem [34]. The trigeminal nerve is the largest cranial nerve that exits the cranium carrying motor innervation to the muscles of mastication and receiving sensory inputs from proprioceptors of the teeth. Based on our previously published study [20, 21], we hypothesize that brain housekeeping molecules may travel from the masticatory muscles to the brain through the trigeminal nerve after stimulation from mastication. Clearance of amyloid-β reduces toxicity and results in maintenance of neuronal health, thereby, possibly preventing cognitive decline.

The action of mastication involves movements of the jaw, translation and rotation. When teeth are biting in functional contacts, mechanoreceptors within the periodontium activate sensorimotor circuits and the signals of feedback and feedforward promote rhythmic jaw movements [34]. In response to repetitive oral motor tasks, the associated cortical network undergoes structural and function change overtime, which is called neuroplasticity [34]. As a result of tooth loss, mass and strength of masticatory muscles decrease, leading to difficulty in chewing while diminished afferent signals reduce neuroplasticity [35]. In addition, with aging, the number of teeth present, occlusal force and masseter muscle thickness all decrease [36]. Complete denture wearers are essentially in a state of masticatory incapacity while removable partial dentures offer only a poor addition to occlusion [37]. After complete denture rehabilitation, studies have observed a subsequent increase in muscle thickness, though remaining thinner than that of dentate individuals [38]. Reduced masticatory ability was found to impair spatial memory and learning ability linked to morphological changes, decreased neurotrophic protein expression and activities in the hippocampus [39].

In the present study, we found the crude odds ratio with a significance level of *p*<0.0001 which strongly supports the hypothesis that a lack of mastication by the natural teeth due to tooth loss may negatively impact cognitive health. Although a removable prosthesis contributes to reproducing some mastication with denture teeth, maintaining all-natural tooth contact is the ideal for obtaining maximum bilateral bite force. In both groupings (A+B+C vs. D+E+F and A vs. F), the odds ratio in advanced elderly groups is smaller than early elderly group. This can be explained by the fact that the bite/occlusal force continuously decreases after age 50 [23, 40].

A major limitation of this study is the lack of information on confounding variables such as population demographics, other systematic diseases, and socioeconomic status. There is also a lack of information regarding other diseases causing cognitive impairment such as stroke, schizophrenia, alcohol, or drug-induced mental disorders. In future studies, known risk factors should be included to eliminate potential bias. However, we were able to detect an association between cognitive function and masticatory condition among elderly adults. Although removable prosthesis wear serves as a marker for tooth loss and a good predictor of cognitive impairment, the association between tooth loss and cognitive impairment was only suggested. Direct causative roles are yet to be proven. Multiple studies have shown the association between the tooth loss and diminished cognitive function, however, there remains conflicting evidence in longitudinal studies regarding the risk of developing incident dementia and tooth loss due to methodological limitations [28, 41]. Future studies using longitudinal data with an extensive period and larger sample size are needed to confirm and test these associations.

To the best of our knowledge, this study is the first to look at a large elderly population diagnosed with multiple stages of cognitive impairment and compare their masticatory conditions. Japan’s UHCS database used for this descriptive analysis covered 33 million patients (age >65) which is more than 94% of the population, and the masticatory condition of 70% of those patients (more than 23 million patients) was investigated. Supplement to the current knowledge [42, 43], our data demonstrate a possible association between oral and cognitive health. The mechanisms of these changes require further elucidation. The implications of this study emphasize the importance of oral health throughout life and the maintenance of natural teeth to allow individuals to have a better quality of life both physically and mentally.

## MATERIALS AND METHODS

### Collection of medical and dental records

**Figure 2:**
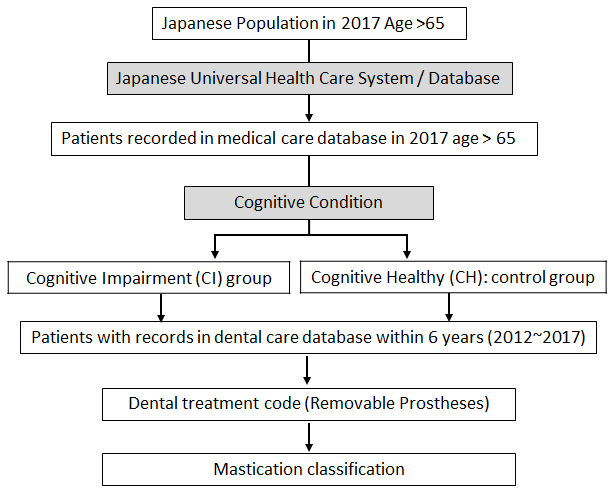
**Scheme of the study data extraction from Japanese universal healthcare system (UHCS) database.**

This study was approved by Japan’s Ministry of Health, Labor and Welfare in 2018, and the dataset is obtained from the Japanese (UHCS) in 2020 and was approved by the Institutional Review Board of Iwate Medical University Japan (IMU_01301). Figure 2 indicated the scheme for data collection. Patients over 65 years old who visited a medical office in 2017 were the first group included in the study. Patients in that group diagnosed with and without cognitive impairment (Alzheimer’s disease or dementia) were identified. Diagnoses include pre-senile dementia of the Alzheimer’s type, Alzheimer’s type dementia, Alzheimer’s type not specified dementia, senile dementia of the Alzheimer’s type, frontotemporal dementia, senile cognition, dementia, cortical dementia, and subcortical dementia. Then among those patients, those who visited the dental office during 2012~2017 and received removable prosthesis were extracted and used for data analysis.

### Classification of mastication

**Figure 3:**
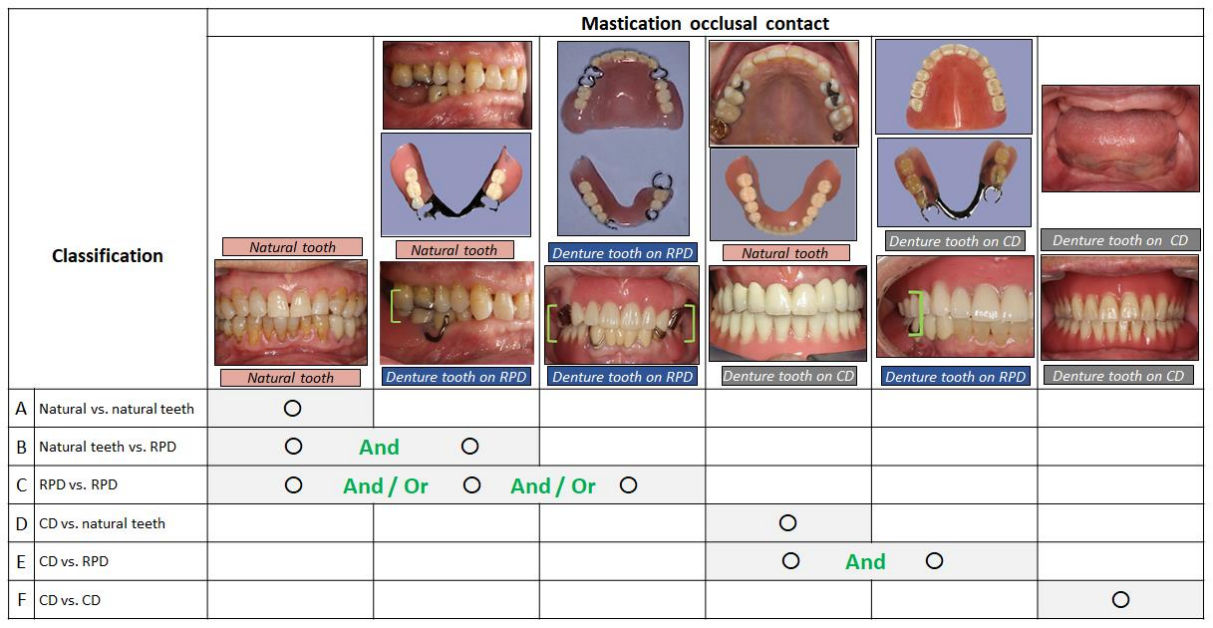
**Mastication classifications and occlusal contact.** RPD design varies depending on the number of missing teeth and classification B, C, and E include multiple scheme of occlusal contact pattern.

The masticatory schemes were determined using dental insurance codes for removable prosthetic treatments; complete dentures (CD): 313017010, and removable partial dentures (RPD). Subjects were then categorized into six groups of masticatory classifications depending on their masticatory patterns: subjects with (A) no removable prostheses (dentures) and only tooth-borne occlusion by natural-natural tooth contact; (B) an RPD in one arch with occlusion of natural-denture tooth contacts in addition to natural-natural tooth contacts; (C) RPDs in both arches with occlusion in combination of denture-denture and/or natural-denture and/or natural-natural tooth contacts; (D) a CD in one arch with occlusion of only natural-denture tooth contacts; (E) CD and RPD in opposing arches with occlusion of denture-denture and denture-natural tooth contacts; and (F) CDs in both arches with only denture-denture tooth contacts in the absence of natural teeth (Figure 3). RPDs vary in shape depending on the number of missing teeth and sample images of RPD are represented in Figure 3.

### Statistical analyses

To analyze the association between cognitive condition and masticatory condition, the six masticatory patterns were compared between the cognitive impairment group (CI group) and the control group (CH: cognitive healthy group). The data was analyzed in two different age groups: 65 to 74 (early elderly, insurance covers 70%~80% of the cost), and those 75 and older (advanced elderly group, insurance covers 90% of the cost). The crude odds ratio was calculated to examine the association between masticatory condition and cognitive health. Six masticatory classifications were categorized into two combinations of grouping. Grouping A: (1) mastication of natural tooth-tooth exists (Classification A+B+C) and (2) mastication of natural tooth-tooth absent (Classification D+E+F) – all mastication is on denture teeth. Grouping B: (1) only natural dentition contacts (Classification A) and (2) only complete denture contacts (Classification F).
